# Internet delivered, non-inferiority, two-arm, assessor-blinded intervention comparing mindfulness-based stress reduction and cognitive-behavioral treatment for insomnia: a protocol study for a randomized controlled trial for nursing staff with insomnia

**DOI:** 10.1186/s13063-022-06986-3

**Published:** 2022-12-16

**Authors:** Yaling Li, Nabi Nazari, Masoud Sadeghi

**Affiliations:** 1 Mental Health Education and Counseling Center, Shenzhen Technology University, Shenzhen, 518118 Guangdong China; 2 Department of Psychology, Faculty of Human Sciences, Lorestan University, Khorramabad, Iran

**Keywords:** Sleep disorders, Insomnia, CBT-I, MBSR, Internet, COVID-19, Treatment

## Abstract

**Background:**

Insomnia and poor sleep quality are highly prevalent conditions related to coronavirus disease 2019 (COVID-19) complications among clinical nurses. Although cognitive behavioral therapy for insomnia (CBT-I) is a first-line treatment, CBT-I suffers from several major drawbacks. This study investigates whether the application of the internet-delivered mindfulness-based stress reduction (iMBSR) intervention will produce effects that are non-inferior to the internet-delivered CBT-I (iCBT-I) intervention in reducing the severity of insomnia in clinical nurses with insomnia at the end of the study.

**Methods:**

This study protocol presents an internet-delivered, parallel-groups, assessor-blinded, two-arm, non-inferiority randomized controlled trial. The primary outcome is sleep quality, assessed by the Insomnia Severity Index. Secondary outcomes include depression, dysfunctional beliefs, five facets of mindfulness, and client satisfaction.

**Conclusion:**

It is expected that this study may address several gaps in the literature. The non-inferiority study design is a novel approach to evaluating whether a standardized, complementary treatment (i.e., MBSR) is as practical as a gold standard treatment rather than its potential benefits. This approach may lead to expanded evidence-based practice and improve patient access to effective treatments.

**Trial registration:**

Trial registration number: ISRCTN36198096. Registered on 24th May 2022.

**Supplementary Information:**

The online version contains supplementary material available at 10.1186/s13063-022-06986-3.

## Introduction

### Background and rationale {6a}

#### Background

The outbreak of coronavirus disease 2019 (COVID-19) is an emotionally challenging time for healthcare workers (HCWs), associated with a substantial increase in the prevalence of physical and mental health problems [1, 2]. For example, the nursing staff is more likely to report for a COVID-19 positive test [3, 4]. Following the COVID-19, the demand for medical personnel surged considerably. The lack of healthcare workers has led to a dramatic increase in work overload, as well as an elevated psychological distress among HCWs [5]. Additionally, numerous studies during the COVID-19 pandemic have suggested that clinical nurses are at high risk of developing depression, anxiety, and insomnia compared with general populations [6–9]. During the COVID-19 pandemic, research shows a high prevalence of pandemic-related sleep disturbance among clinical nurses [4, 10, 11].

Insomnia is a highly prevalent complaint in primary care settings and it is characterized by difficulties in sleep initiation or maintenance, which occur at least three times per week for less than 3 months [12]. The consequences of insomnia range from loss of productivity, absenteeism, and job accidents [13] to cognitive performance [14]. Also, insomnia has bidirectional causation and interactive relationships with anxiety [15], depression [16], and difficulties with emotion regulation [17]. For clinical nurses, insomnia impact of quality of life [18], job satisfaction and the care of patients [19], work performance [20], immune system [21], and the turnover intention [22]. Therefore, it is extremely important to reduce the severity of insomnia symptoms among nurses during the pandemic. However, there have been insufficient preventative interventions on clinical nurses’ present sleep disruption status. Despite the prevalence and clinical significance, insomnia is frequently underdiagnosed [23] and, consequently, undertreated. Untreated insomnia carries the burden of added lower job productivity and quality of life [5, 24] and physical health risks, such as cardiovascular disease [25], stroke [26], hypertension, and diabetes [27]. Insomnia is a highly prevalent problem related to COVID-19 complications for nurses [7]. During the current pandemic, nurses may be at a higher risk of mental health problems than doctors [28]. Being a frontline nurses is independently associated with higher levels of depression, anxiety, and insomnia [29]. In comparison to non-medical HCWs, HCWs in direct contact with COVID-19 patients had significantly higher levels of insomnia, anxiety, and depression [30]. Additionally, contact with COVID-19 patients is independently associated with elevated risk of sleep disturbance [31]. Although addressing the needs of nurses is a high priority, information to inform such contributions is limited.

The pharmacologic treatments for insomnia have side effects and increase dependency [32]. Therefore, psychological interventions must be immediately implemented to promote the mental health of nurses exposed to COVID-19. Among non-pharmacological treatments, CBT-I is a multi-component intervention for insomnia and is recommended as first-line therapy [33]. The CBT-I addresses a wide variety of psychological issues, from modifying cognitive beliefs [34] to reducing psychiatric symptoms [35, 36]. CBT-I effectiveness for the treatment of insomnia comorbid with other disorders has been also well-documented [37–40]. Although CBT-I is a low-risk, productive, and durable treatment, some drawbacks are related to effectiveness and accessibility.

Regarding effectiveness, a significant number (30 to 40%) of insomnia patients did not respond or remit, and over half (50–60%) experienced a varying degree of insomnia symptoms following a CBT-I intervention [41]. One potential explanation is poor adherence to treatment [42]. Several core components of CBT-I (e.g., sleep restriction and stimulus control) can be difficult to implement and often result in a short-term worsening of symptoms and patient discomfort [43]. While CBT-I is effective for the treatment of insomnia comorbid with depression, this intervention may not adequately address the numerous factors that contribute to the development and maintenance of insomnia [35]. Regarding accessibility, main causes of limited access to insomnia treatments are identified. First, despite significant attempts to expand the numbers of CBT-I qualified experts the accessibility of CBT-I is still mostly restricted to clinical settings or major cities [44]. Second, the CBT-I requires a highly qualified therapist who is not geographically available. In addition to the lack of CBT-I therapists, professionals are uninformed of available therapy options. Finally, insurance payment for CBT-I may be inadequate, especially if it is provided by mental health professionals who are not physicians (such as psychologists). These barriers highlight the need for approaches that could be implemented more easily in public. Clearly, there is a need to improve and expand the available insomnia treatment options. Mindfulness-based interventions (MBIs) are additional therapies that may solve this drawback of CBT-I. A complementary and supplementary treatment, which can address the CBT-I limitations, incorporates mindfulness.

Mindfulness is a mind’s awareness that emerges through deliberate concentration of the moment, in the present moment, and on non-judgmentally moment-by-moment experience with an attitude of curiosity, openness, and acceptance [45, 46]. MBIs are the type of non-pharmacological psychotherapy that has grown dramatically in recent years for several health-related disorders, including sleep problems and insomnia [47–50]. The MBIs effects are strong in studies primarily aimed at improving sleep [51]. Mindfulness techniques help individuals recognize earlier and more skillfully respond to the chronic or maladaptive patterns of mind. MBIs aim to directly reduce pre-sleep arousal and may avoid the short-term unpleasant consequences sometimes seen in CBT-I [52].

Clinical nurses also experience other symptoms comorbid with sleep disturbance, which CBT-I may not target, such as distress generated by stressful events [53] and difficulty with emotion regulation [54]. During the COVID-19, nursing staff experienced a great deal of physical and mental health issues, such as fatigue, sleep disorders, and burnout. Having to tell patients bad news is one of the difficult yet inevitable part of the medical profession. To maintain their own emotional equilibrium and mental wellbeing, they must be provided with the skills and training to be able to regulate such intense emotions. Mindfulness is related to positive emotion and life satisfaction [55], job productivity, and well-being [56]. Higher mindfulness levels are negatively associated with anxiety, psychological distress, and depression [57, 58]. For nurses, mindfulness is related to job performance, job satisfaction, and employee well-being [59]. Regarding coping with stressful events, the promotion of emotion regulation skills is suggested to underlie the benefit of mindfulness [60, 61]. One randomized controlled trial has compared an MBI to CBT-I [62]. The study revealed no significant differences in long-term efficacy for reducing insomnia severity. An online group mindfulness training program showed that mindfulness training via videoconferencing may be an effective intervention for stress reduction during the COVID-19 pandemic [63]. The “CoPE It” programs, E-mental health mindfulness-based and skills-based and consists of techniques of MBSR, help in reducing psychological distress in times of COVID-19 [64].

While individuals have the above-mentioned difficulties in accessing an appropriate insomnia intervention, at the same time, COVID-19 instructions and restricting policies (e.g., physical distancing) to minimize the risk of infection have made more difficult to seek and attend treatment. The Internet delivery of mental health services is a technological revolution that offers the possibility of expanding more accessible and cost-effective psychological interventions [65]. Meta-analytic findings have shown that Internet-based therapies are effective for insomnia [66, 67]. Recent research has demonstrated that Internet-based cognitive-behavioral therapies are an efficient and promising approach for various conditions, such as insomnia [68]. Compared with traditional face-to-face interventions, internet-delivered interventions provide widespread access and dissemination and increase cost-effectiveness [69, 70].

### Objectives {7}

The objective of this study is:

1. To investigate whether the application of the internet-delivered MBSR intervention will produce effects that are not inferior to those of the internet-delivered CBT-I intervention in reducing the severity of insomnia in clinical nurses with insomnia at the post-intervention (8 weeks)
2. To examine the effectiveness of an iCBT-I intervention to improve insomnia symptoms, dysfunctional beliefs and attitudes about sleep, depressive symptoms, mindfulness aspects, and quality of life in nurses
3. Investigate whether internet-delivered MBSR intervention could improve insomnia symptoms, dysfunctional beliefs and attitudes about sleep, depressive symptoms, mindfulness aspects, and quality of life in clinical nurses

### Trial design {8}

**Fig. 1:**
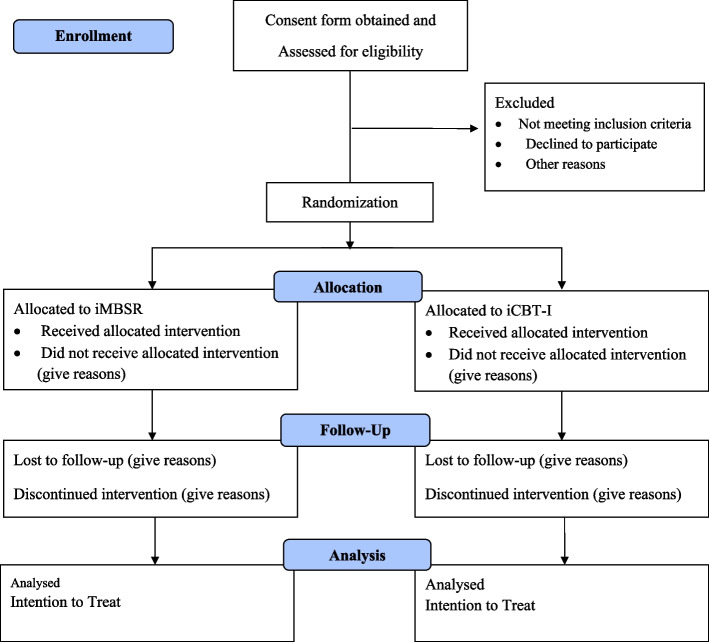
The consort flow diagram

This study is designed as a non-inferiority randomized controlled, Internet-delivered, parallel-group, assessor-blinded, two-arm trial comparing iCBT-I with an iMBSR. The study flowchart is displayed in Fig. 1. The SPIRIT (Standard Protocol Items: Recommendations for Intervention Trials) checklist of information was used to report a randomized trial [71] (see supplemental data: appendix 1).

## Methods: participants, interventions, and outcomes

### Study setting {9}

Participants will be recruited between June 2022 and August 2022 in Iran. Ethical approval for the study was obtained from the Research Ethics Committees of Lorestan University of Medical Sciences (reference number: IR.LUMS.REC.1399.269). The trial has been registered at (ISRCTN36198096). The study was found to be in accordance with the national norms per the Helsinki Declaration.

### Eligibility criteria {10}

The nurses who are included in the care of COVID-19 patients will be recruited in this study.

The inclusion criteria are:

- Age ≥ 18 years
- Insomnia Index Scale ≥ 15
- Willing to participate
- Access to the Internet and have an email address
- Meeting the *Structured Clinical Interview for DSM-IV Axis I Disorders* criteria for insomnia [72, 73].

*Exclusion criteria:*

- Receiving psychological interventions during this study
- Pregnancy or breastfeeding
- Missed three consecutive sessions

#### Who will take informed consent? {26a}

**Fig. 2:**
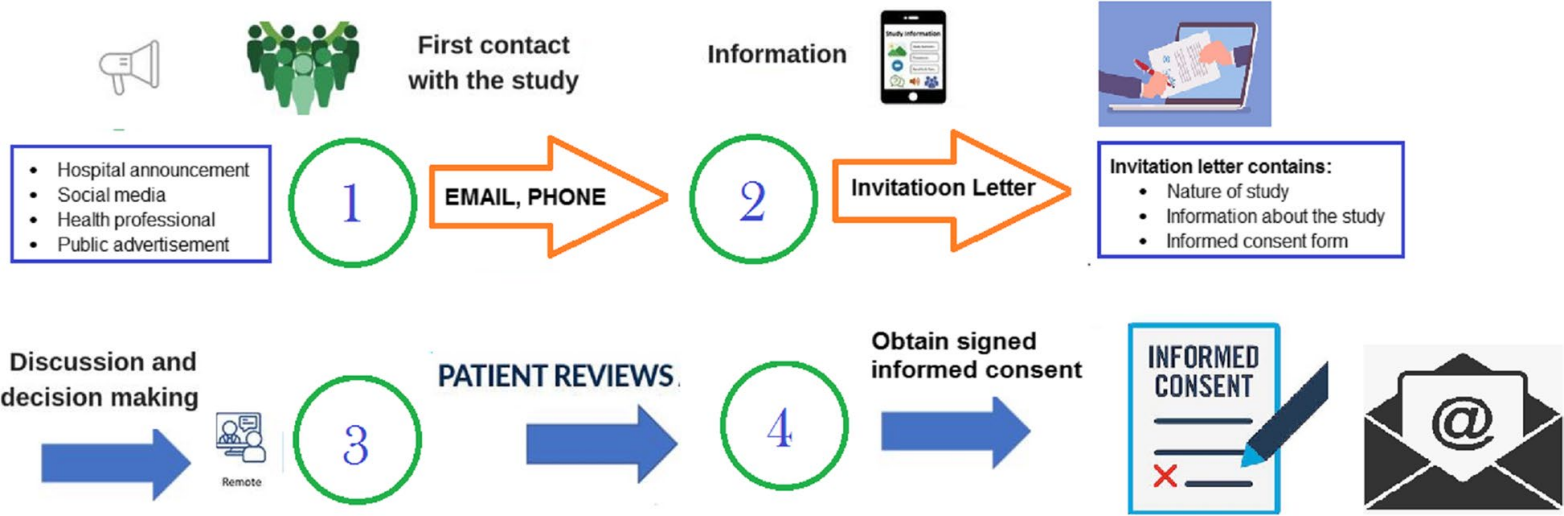
Graphical model of obtaining informed consent form process

Participants will be recruited through online announcements, flyers, and referrals (see Fig. 2). Potentially eligible individuals can apply to participate in the RCT via email or telephone contact. Interested participants will be informed of the study’s objectives, advantages, hazards, session numbers, confidentiality, and assurances of anonymity via email or telephone. Participants will be informed that they will be free to withdraw their consent or decline to participate. Upon agreement to participate, a survey invitation letter will be sent via email. Once the participants clicked on the survey link, an informed consent page will be opened. The nurses should be obtained an electronically signed informed consent form to proceed with the instrument section. Individuals who initially obtain a high score on the Insomnia Severity Index (ISI ≥ 15) will undergo an interview to ensure that the eligibility criteria are fulfilled. Two clinical psychologists evaluated the participants' personal history, mental status, personal resources, and suicide risk through 45 min of online clinical interviews.

#### Additional consent provisions for collection and use of participant data and biological specimens {26b}

Nurse will be requested if they agree to be contacted in the secondary analysis in future research participation. No biological specimens were collected as part of this trial.

### Intervention description {11a}

#### Interventions

The study (e.g., all assessments and treatments) will be administered online. All participants s will be requested not to change their lifestyle during the study and continue their daily routines, exercise, diet, and medications, and, if received any psychological treatment, inform the researchers. Each participants who receive positive COVID-19 test will be exempted to continue the trial. Before each session, participants will be requested to feel free in case of unpleasant experiences, potentially related to the trial. In case of emergency, the participant will receive further evaluation to make decision about continue or discontinue.

#### IMBSR group

The iMBSR intervention will be delivered in eight 2.5-h weekly sessions [74, 75], plus one 6-h weekend intensive silent retreat. The program is intended to facilitate increased awareness of one’s typical internal and external reactions to stress and introduce meditation techniques to promote healthy responses to stress. The core components of the IMBSR program comprise: (*a*) motivational interview; (*b*) physiological and psychological response to go; and (*c*) engage in a combination of the three major formal mindfulness practices (mindful hatha yoga sitting meditation and the body scan); (*d*) information on the effect of stress on health and options for mindful versus reactive responses to stress including non-judging, acceptance, patience, non-striving, and letting; and (*e*) course overview and relapse prevention. The program will be started with a module on motivation and readiness for change and treatment engagement and will be finished with a module for relapse prevention (for a more thorough explanation, see the supplemental data: appendix 2: part two).

#### ICBT-I group

The CBT-I group format is typically delivered over six to eight sessions [76–78]. The CBT-I is a multi-component intervention that will be administered and structured based on eight 2-h weekly sessions. The core components of the ICBT-I program comprise the following: (*a*) motivational interview, (*b*) sleep hygiene education; (*c*) sleep restriction; (*d*) stimulus control, (e) cognitive therapy, and (*f*) course overview and relapse prevention (for a more thorough explanation, see the supplemental data: appendix 2: part one).

#### Strategies to improve adherence to interventions {11c}

The motivational interview will be delivered to maximize the participants’ readiness for change and increase motivation to engage in treatment. Written feedback will also be provided to all nurses about the results of the health screenings. Reminder and follow-up messages will be sent to participants within three days before and after the each time-points

#### Relevant concomitant care permitted or prohibited during the trial {11d}

All standard care and stable medication are permitted other than concomitant administration of any other experimental treatment (see exclusion criteria).

#### Provisions for post-trial care {30}

No special provisions are offered.

## Outcomes {12}

The survey comprised the Dysfunctional Beliefs and Attitudes about Sleep Scale (DBAS) [79]), Brief Patient Health Questionnaire-9 (PHQ-9 [80]), Insomnia Severity Index (ISI [81]), The Five Facet Mindfulness Questionnaire (FFMQ [82]), and Client Satisfaction Questionnaire Adapted to Internet-Based Interventions (CSQ-I [83]).

### Primary outcome

The ISI is a brief scale employed to measure sleep problems and the severity of insomnia. This scale is sensitive to treatment response, which yields 86.1% sensitivity and 87.7% specificity [84]. Respondents will endorse seven items on a five-point scale ranging from zero = no problem to four = very severe, with higher scores indicating worse insomnia. ISI score ≥ 15 will be found to represent the moderately to severe clinical insomnia.

### Secondary outcome

#### Dysfunctional beliefs

DBAS is employed to measure erroneous beliefs, impaired cognition, expectations, and dysfunctional attitudes regarding poor sleep. Respondents will endorse ten items on Likert scales ranging from 0 (strongly disagree) to 10 (strongly agree).

#### Mindfulness

The FFMQ is a self-administered questionnaire employed to measure the five aspects of mindfulness. The questionnaire comprises five facets, including observation, description, aware action, nonjudgmental inner experience, and non-reactivity. The scale consists of 15 items (a three-item for each factor) that are rated on a five Likert scale from 1 (never) to (very often).

#### Client satisfaction

CSQ-I is an eight-item self-report instrument adapted to measure participant satisfaction with internet-delivered interventions. Nurses rate each item on a four-point Likert scale ranging from “does not apply to me” to “applies to me.”

#### Adherence

Adherence can be conceptualized as the extent to which individuals experience or engage with the content of an online intervention. Adherence is measured by checking the number of modules completed in ICBT-I and group sessions attended in IMBSR. The feasibility of the both online interventions will be computed by the completion rate of each session and the proportion of participants completing the study.

### Participant timeline {13}

**Fig. 3:**
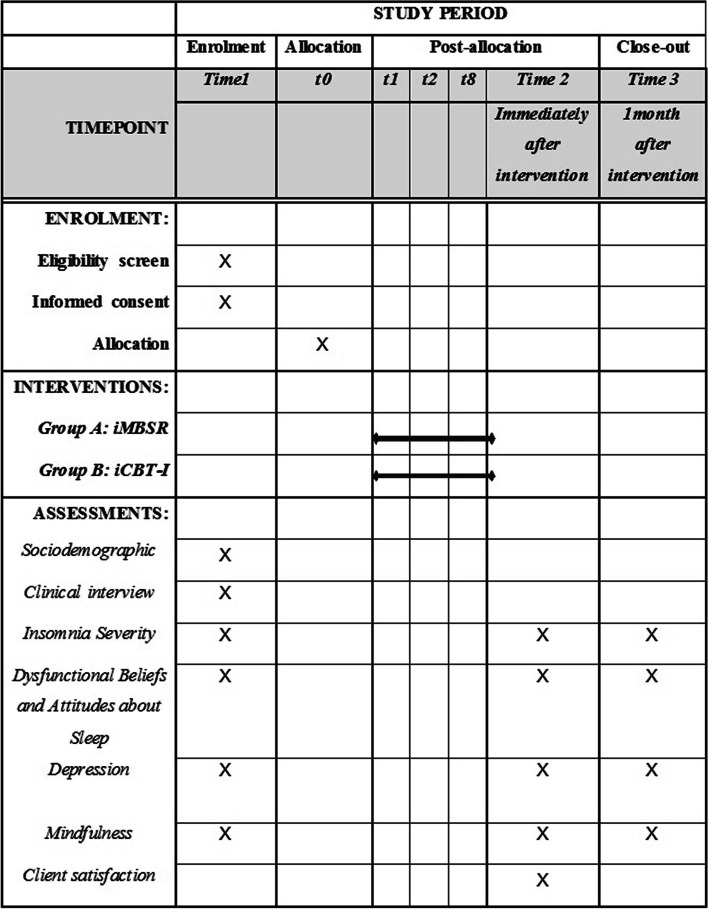
Study schedule of enrolment, intervention, and assessments

Study schedule of enrolment, intervention, and assessments is presented in Fig. 3.

### Sample size {14}

The sample size is calculated according to the minimal difference recommendation for non-inferiority design [85]. The minimal important difference in the severity of insomnia is a decrease of eight scores on the ISI [84]. The non-inferiority margin is half the difference (4 points on ISI). Therefore, the treatment groups will be considered equivalent if their average ISI total scores do not differ by more than 4 points. Therefore, the study will detect the minimum clinically significant difference at a one-tailed test, 5% significance level, and 80% power. Finally, 64 participants will be required. Anticipation of 25% dropout rate, the required sample size was 80. To reach the desired sample size, the link for the study will be distributed and posted on online medical staff forums. Also, the trial information will be emailed to psychologist and psychiatrist to share the trial with their clients.

### Randomization and masking {16a}

Participants will be randomly allocated to either of both conditions, using the permuted block technique. An independent statistician will generate the allocation schedule without any influence of the principal investigators or therapists. Random sequence block sizes (i.e., 2, 4, and 6) will be generated using a computer random number generator with equal allocation ratio (1:1). The statistician informs the observer staff and participants of the random allocation results via email. Randomization will be computerized with a concealed randomization. Block size will be unknown to the investigators and clinicians. The randomized intervention allocation is concealed until the statistical analyses of the resulting data will be completed and outcomes will be drawn. The psychological evaluators, statisticians, and assessors who measure and record the data will be blinded to the conditions and the participants’ groups. The research team will not be able to influence the randomization sequence and will have no access to allocations. The participants will be blinded to the study hypotheses.

The data safety and monitoring committee may request unblinded data throughout the trial period, but the initial data analyses should be blinded to protect against bias in decision. The investigator must report all code breaks (with reason) as they occur on the corresponding case report form page. Unblinding should not necessarily be a reason for study discontinuation.

### Data management {19}

The data will be collected via the Internet. The participants will complete the primary and secondary outcomes at three-time points: time 1: pretreatment to allocation, including baseline (week 1); time 2: immediately after the intervention, including post-treatment assessment (week 10), and time 3: 1-month follow-up (week 14). The data will be automatically coded and depersonalized and stored on a secure server. Before recruitment, the study protocol was designed with a concentrate on ensuring data quality through standardization of data collection processes. Data collection instruments were designed with validated items. Also, instrument guides were utilized as a reference for data collectors and supervisor, which included instructions for how to use instruments, definitions, and interpretation guidelines for each question. Also, all research team members received a comprehensive refresh course in the context of psychological assessments and ethics in clinical research. The supervisor ensure that each operation will be performed to a standard.

### Confidentiality {27}

All trial-related data will be coded and depersonalized and will be securely stored using encrypted digital files within password-protected folders. The data accessibility will be limited to the principal investigator and monitoring board. Monitoring member is full professor of psychology and high experienced researcher. All data will remain confidential before and during the trial.

## Statistical methods

### Statistical methods for primary and secondary outcomes {20a}

Main analyses will be conducted on the intent-to-treat (ITT) approach, including all randomly allocated participants, regardless of attendance. The last observation carried forward (LOCF) method is one way to handle missing data. In LOCF approach, the missing data are replaced by the last observed value of that variable. The time points for the observations of missed values will be reported. Descriptive statistics will be utilized to present the continuous variables as the means and SDs and categorical variables as the numbers and percentages.

To determine whether the IMBSR is inferior to ICBT-I, the confidence interval approach was used. Non-inferiority analysis will be assessed using an *F* test statistic generated from the linear mixed model and confidence intervals [86]. This tests whether the difference between the group means is statistically larger than the non-inferiority margin (clinically significant difference) and if the upper limit of the one-sided confidence interval of the difference between the group means is less than the pre-specified margin of non-inferiority. A non-inferiority margin of 4 points on ISI is chosen for the analysis. This means that the mean difference between the groups should not be more than 4 points in favor of ICBT-I, to establish noninferiority of IMBSR. Repeated-measures analyses of variance (ANOVAs) two (condition: iMBSR *vs*. iCBT-I) × three (time point: time1, time2, time3) will be designed to compare the main treatment effects of the conditions on secondary outcomes. The values of effect sizes are presented as partial eta squared and Cohen’s *d.*

### Interim analyses {21b}

Not applicable as no ancillary studies are performed.

### Harms {22}

Despite few adverse effects that have been reported for the both interventions in many trials, the adverse effects in the study will be recorded and reported to sponsor and IRB. All intended and unintended results will also be published. The 24 h/7day phone number will be provided to report adverse effects. Participants will be told to feel free to contact us when needed.

### Dissemination plans {31a}

The study’s findings will be disseminated in publication with a double peer-reviewed policy. The dataset will be made available to academic investigators upon request from the corresponding author. Before a formal amendment to the protocol, any minor or major changes or modifications to the protocol which may lead to substantiate impacts on the study (i.e., study objectives, study design) or may affect on the benefit or safety of the participants or methodology (i.e., sample sizes) must be approved by the IRB. The template informed consent forms will be reviewed and approved by the IRB (see supplementary data).

### Plans, if any, for granting public access to the full protocol, participant-level dataset, and statistical code {31c}

No later than 1 year after the termination of the study, a completely de-identified deliver data set will be submitted to ISRCTN and publicly available.

## Discussion

The outbreak of COVID-19 is an emotionally challenging time, especially for nurses, and is associated with a range of mental and medical health problems with long-lasting effects. Although addressing the needs of front-line health care workers during the COVID-19 pandemic is a high priority, evidence-based evaluations and mental health interventions targeting front-line health care workers are relatively scarce. Special interventions to promote mental well-being in health care workers exposed to COVID-19 need to be immediately implemented, with nurses, and frontline workers requiring particular attention. Internet-delivered interventions can effectively prevent the progression of insomnia and improve comorbid symptoms and quality of life among nurses. It is expected that MBSR can effectively reduce insomnia symptoms. Individuals with depression experience deficits in mindful awareness of their emotions and experiences, particularly for nonjudgmental awareness and allowing oneself to notice emotions without triggering a repetitive negative thinking process such as rumination [87]. Mindfulness training is associated with decreased reliance on strategies such as rumination and over engagement and a more remarkable ability to tolerate negative emotions [88, 89]. It is expected that this study may address several gaps in the literature. Application of MBIs for insomnia may serve as a potential alternative or supplement to CBT-I.

The principal limitation of this present trial is the absence of placebo intervention. Additionally, although semi-structured insomnia interviews will be conducted to identify sleep disorders, patients are not screened for sleep apnea or periodic limb movement disorder. Additionally, with short-time follow-up, determining long-term effects will be difficult. Yoga has been shown to improve sleep. In this study, we do not have a plan to measure the difference between the effects of yoga exercises and IMBSR psychological exercises. Measuring the impact of each of these exercises can be considered in future studies.

## Conclusions

Despite limitations, the non-inferiority study design is a novel approach to evaluating whether a standardized, complementary treatment (i.e., MBSR) is as practical as a gold standard treatment rather than its potential benefits. This approach may lead to expanded evidence-based practice and improve patient access to effective treatments. Additionally, both interventions will be delivered through the Internet. Therefore, participants are not limited by environmental, geographical, medical, and mental issues, which will increase the generalizability of the results. Finally, establishing the ability of a standard manualized treatment such as MBSR to improve psychological distress and insomnia positively will increase available treatment options across healthcare workers and clinical nurses with insomnia. COVID-19 has limited availability of face-to-face interventions. While the application of the new technology in health settings was considered a far-reaching goal, the pandemic has acted as a catalyst to progress the use of the new technology in psychiatric settings. Consistent with this trend, treatment issues related geographical distribution of expert is now less divesting. Online CBT-I, electronically delivered intervention, and mobile health application course online represent a potential solution to overcome several of the barriers and could be a viable alternative method for mental health care delivery. The empirical studies suggest MBI can improve sleep quality in a diverse of clinical populations with sleep disturbance [90–93]. In addition to stress reduction, mindfulness-based interventions can also enhance nurses’ capacity for focused attention and concentration by increasing present moment awareness [94], while several evidence indicates that mindfulness meditation improved sleep quality compared with nonspecific active controls [95]. The study’s findings may provide greater confidence that the potential benefits of MBIs, which are not attributed to placebo effects commonly observed in usual care and waitlist controlled trials.

## Trial status

P**a**rticip**a**nt recruietment will be started on June 2022. It is expected that enrolment will continue until July 2022. Ethical approval for the study was obtained from the Research Ethics Committees of Lorestan University of Medical Sciences (reference number: IR.LUMS.REC.1399.269).

## Supplementary Information

**Additional file 1: Appendix 1.** Cognitive behavioral treatment for insomnia program. **Appendix 2.** Mindfulness based stress reduction program.

**Additional file 2.**

## Data Availability

Not applicable.

## Abbreviations

CBT: Cognitive behavioral therapy
CBT-I: Cognitive behavioral therapy for insomnia
COVID-19: Coronavirus disease 2019
CSQ-I: Client Satisfaction Questionnaire Adapted to Internet-Based Interventions
DBAS: Dysfunctional Beliefs and Attitudes about Sleep Scale Questionnaire
DSM: Diagnostic and Statistical Manual of Mental Disorders
FFMQ: Five Facet Mindfulness Questionnaire
HCWs: Healthcare workers
ISI: Insomnia Severity Index
ITT: Intent-to-treat
MBIs: Mindfulness-based interventions
MBSR: Mindfulness-based stress reduction
PHQ-9: Patient Health Questionnaire
RCT: Randomized controlled trial
SCID- IV –I: Structured Clinical Interview for DSM-IV Axis I Disorders
SPIRIT: Standard Protocol Items: Recommendations for Intervention Trials
