# Supplementary material for: Internet delivered, non-inferiority, two-arm, assessor-blinded intervention comparing mindfulness-based stress reduction and cognitive-behavioral treatment for insomnia: a protocol study for a randomized controlled trial for nursing staff with insomnia
Source: Trials. 2022 Dec 16;23:1020. doi: 10.1186/s13063-022-06986-3 (PMC9756716; doi:10.1186/s13063-022-06986-3)
Supplement: Supplementary file 1 — Additional file 1: Appendix 1. Cognitive behavioral treatment for insomnia program. Appendix 2. Mindfulness based stress reduction program. [file 13063_2022_6986_MOESM1_ESM.pdf]

Appendix 1:

Cognitive behavioral treatment for insomnia program:

Session1: Introduction

1) Maximize the patient's readiness for change and increase motivation to engage in treatment.
2) Treatment goal setting and Help patients clarify their goals for treatment and to explore the benefits changing versus staying
3) Discuss the overview of the CBT-I program and the basic principles of how to cope with sleep problems.
4) Help participants set realistic goals
5) Provide introduction/orientation to CBT-I
6) Discuss about the importance of mastering the technique of sleep consolidation.

Session 2: Sleep basic

1) Review of sleep diary (and address any concerns regarding self-monitoring)
2) Discuss the importance of self-monitoring.
3) Psychoeducation about the basic biology of sleep and to become familiar with terms such as sleep efficiency, sleep stages, and slow-wave sleep
4) Presentation of sleep hygiene recommendations
5) Presentation of rationale and strategies for increasing physical activity
6) Rationale anchored in sleep regulation theory and Spielman et al.'s model of insomnia
7) Identify potential obstacles to adherence and address them

Session 3: Stimulus control

1) Review progress using sleep consolidation procedures and help adjust the sleep window.
2) Introduce stimulus control/sleep restriction therapy guidelines.
3) Explain cognitive arousal and its role in maintaining insomnia
4) Review of sleep diary; adjustment of sleep window
5) Continuation of stimulus control and sleep restriction procedures

Session 4: Cognitive therapy

1) Review of problems encountered during implementation of stimulus control and sleep restriction
2) Discuss self-defeating thoughts, negative feelings, and poor sleep behaviors.
3) Discuss the general techniques for managing stress.
4) Identify and challenge participants' negative self-talk about their sleep.

Session 5:

1) Review of sleep diary;

2) Review biased ways of thinking about sleep, signs that self-talk may be negative and self-defeating and the categories of negative self-talk.
3) Examine attitudes and beliefs about sleep.
4) Introduction to brief cognitive therapy for insomnia
5) Continuation of stimulus control and sleep restriction procedures
6) Follow-up on brief cognitive therapy.

Session 6 & session 7:

1) Review of sleep diary;
2) Review biased ways of thinking about sleep, signs that self-talk may be negative and self-defeating and the categories of negative self-talk.
3) Examine attitudes and beliefs about sleep.
4) Follow-up of stimulus control and sleep restriction procedures
5) Follow-up on cognitive therapy.

Session 7

1) Overview of significant treatment concepts
2) To evaluate the patient's progress and to plan for the future.
3) Reinforce the skills learned in treatment, review key treatment concepts
4) Help patients develop strategies for preventing "relapse."
5) Address symptom recurrence
6) Prevention of recurrence, an overview of treatment concepts
7) Discussion about patient's healing and progress and plans for future practices and how patients can maintain treatment gains in the long term.

Appendix 2:

Mindfulness based stress reduction program:

Session 1:

1) Maximize the patient's readiness for change and increase motivation to engage in treatment.
2) Help patients clarify their goals for treatment and to explore the benefits changing versus staying
3) Treatment goal setting.
4) Theoretical underpinnings of mindfulness within the context of Mind-Body.
5) Application of self-regulatory skills related to the individual's condition
6) The participant/patient is experientially introduced to mindful eating, some standing yoga stretches, mindfulness of breathing and the body scan meditation.
7) Learn to become familiar with mindful awareness of the body

Session 2:

1) Experiential mindfulness straining and skill development
2) The role of perception and conditioning in the appraisal and assessment of stress.
3) The pivotal role of self-responsibility in the positive development of short
4) Long-term changes in health and health-enhancing behaviors is introduced.
5) Home practice is assigned with an emphasis on the regular daily practice of the body scan introduction of short periods of sitting meditation,
6) The application and integration of mindfulness into the participant's everyday life

Session 3:

1) Practice Formal mindfulness practices -- mindful hatha yoga (ending with a brief body scan), sitting meditation and optional meditation.
2) Inquiry into and exploration of participants' experiences with in-class and assigned home practices.
3) Challenges and insights encountered in formal practice and in integrating mindfulness into everyday life.

Session 4:

1) Engage in a combination of the three major formal mindfulness practices, including : Mindful hatha yoga, sitting meditation and the body scan.
2) Practice brief guided body scan
3) Emphasizes the development of concentration qnd embodiment.
4) Emphasizes the development of the capacity to train and re-direct attention, and the systematic expansion of the field of awareness

SESSION 5:

1) Engage in a combination of the formal mindfulness practices
2) Emphasizes the capacity of participants to adapt more rapidly and effectively to everyday challenges and stressors.
3) Experiential practice emphasis on *responding* (vs. reacting) to stressors.

4) the value and utility of mindfulness in learning to stop, step back and see more clearly/objectively
5) A central element of the session is oriented around the participant's capacity to *recover more rapidly* from stressful encounters when they occur.
6) emphasis on the growing capacity to attend more precisely to a variety of physical and mental perceptions and to use this awareness as a way of deliberately interrupting and intervening in previously conditioned, habitual behaviors and choosing more effective mindfulness-mediated stress responses.

Session 6 & session 7:

1) Experiential training in MBSR
2) Emphasis on the growing capacity to self-regulate and cope effectively with stress.
3) Emphasizes the development of concentration, embodiment,
4) Develop, train and redirect attention, and the systematic expansion of the field of awareness.
5) Engage in an in-depth exploration of stress within the domain of communications
6) Discussion development of "transformational coping strategies "including:

- Awareness, attitudes
- Behaviors that enhance psychological characteristic: "stress hardiness" or resilience.
- Discuss about effective ways to cope with relapse, especially in the context of COVID-19.

Session 8:

1) Overview of significant treatment concepts
2) To evaluate the patient's progress and to plan for the future.
3) Reinforce the skills learned in treatment, review key treatment concepts
4) help patients develop strategies for preventing "relapse."
5) Address symptom recurrence
6) Prevention of recurrence, an overview of treatment concepts
7) Discussion about patient's healing and progress and plans for future practices and how patients can maintain treatment gains in the long term.
