# Supplementary material for: Internet delivered, non-inferiority, two-arm, assessor-blinded intervention comparing mindfulness-based stress reduction and cognitive-behavioral treatment for insomnia: a protocol study for a randomized controlled trial for nursing staff with insomnia
Source: Trials. 2022 Dec 16;23:1020. doi: 10.1186/s13063-022-06986-3 (PMC9756716; doi:10.1186/s13063-022-06986-3)
Supplement: Supplementary file 2 — Additional file 2. [file 13063_2022_6986_MOESM2_ESM.pdf]

Consent to Participate in Research

Invitation to be Part of a Research Study

You are invited to be part of a research study. This consent form will help you choose whether or not to participate in the study. Feel free to ask if anything is not clear in this consent form.

1. I know that the objectives of this research are:

- reducing Insomnia symptoms
- Training in coping skills with sleep disturbances

2. I know that my participation in this research is completely voluntary and I do not have to participate in this research.

I was assured that if I refused to participate in this study,

3. I know that even after agreeing to participate in the research, I can whenever I want, after informing the facilitator, or without informing the facilitator

I will leave the research and my withdrawal from the research will not deprive me of receiving the usual medical services.

4. The way I collaborate in this research is as follows:

- Participate in regular meetings
- complete forms and questionnaires

5. The potential benefits of my participation in this study are as follows:

- Increasing the knowledge and skills that will help me continue to live more peacefully and overcome my Sleep problems.

6. About the harms and possible side effects of participating in this study I was told that: So far no harm has been done to these trainings not reported.

7. I know that those involved in this research have kept all information about me confidential and are only allowed Only publish the general and group results of this research without mentioning my name and details.

8. I know that the Research Ethics Committee can access my information to monitor my rights.

9. I know that I will not incur any of the costs of conducting research interventions as follows.

- Participate in sessions
- Presenting the results of interviews and personal results
- Counseling during the design and diagnostic interviews

10. Mr....... **M.S**............. was introduced to me for a pass and I was told that whenever there is a general problem or question

The relationship with participating in the mentioned research happened to me to share with them and ask for guidance.

11. I know that if during and after the research any problems, both physical and mental, due to participation in this research for me before the treatment of complications, and its costs and related compensation will be the responsibility of the executor.

12. I know if I have any problems or objections to those involved or the research process, I can contact the Research Ethics Committee of the Lorestan University of Medical Sciences.

13. This form of information and informed consent is set in two versions and after signing one copy is available to me and the other version in
Will be at the discretion of the executor.

14. Approved Code of Ethics:

Approval Number: Approval ID: **(reference number: IR.LUMS.REC.1399.269)**

Board Name: **Lorestan University of Medical Sciences**

Data Monitoring: Yes

I read and understood the above
This form was also read to me in the presence of Mr. / Mrs.... as a witness.
After reading the form and at the time of signing, none of the elements of the plan had a physical presence.

At the time of signing this form, I had enough time to decide and I did not have any time limit for signing

Based on that, I express my informed consent to participate in this research.

Participant's signature

I ……………… oblige myself to fulfill the obligations related to the executor in the above provisions and undertake to provide salaries and

The safety of the participant in this study.

Stamp and signature of the researcher
